# Introducing Potential Key Proteins and Pathways in Human Laryngeal Cancer: A System Biology Approach

**Published:** 2018

**Authors:** Hassan Peyvandi, Ali Asghar Peyvandi, Akram Safaei, Mona Zamanian Azodi, Mostafa Rezaei-Tavirani

**Affiliations:** a *Hearing Disorders Research Center, Shahid Beheshti University of Medical Sciences, Tehran, Iran. *; b *Proteomics Research Center, Shahid Beheshti University of Medical Sciences, Tehran, Iran.*

**Keywords:** Human laryngeal cancer, PPI network analysis, Biomarker panel, Cytoscape

## Abstract

The most common malignant neoplasm of the head and neck region is laryngeal cancer which presents a significant international health problem. The present study aims to screen potential proteins related to laryngeal cancer by network analysis to further understanding disease pathogenesis and biomarker discovery. Differentially expressed proteins were extracted from literatures of laryngeal cancer that compare proteome profiling of patient›s tissue with healthy controls. The PPI network analyzed for up and down regulated proteins with Cytoscape Version 3.4. After PPI construction, topological properties of the two networks have been analyzed. Besides, by using MCODE. the Gene Ontology (GO) analysis, the related modules and pathways were examined. Our study screened 275 differentially changed proteins, including 136 up- and 139 down-regulated proteins. For each network, it has been considered 20 key proteins as hub and 20 as bottleneck. A number of 26 hub-bottleneck nodes is introduced for the two networks. A total of 11 modules including 6 downregulated and 5 upregulated network modules were obtained. The most significant GO function in the significant upregulated module was the RNA processing, and the most significant one in the downregulated module with highest score was the respiratory electron transport chain. Among 275 investigated proteins, 12 crucial proteins are determined that 4 of them can be introduce as a possible biomarker panel including YWHAZ, PPP2R1A, HSP90AA1, and CALM3 for human laryngeal cancer.

## Introduction

The most common malignant neoplasm of the head and neck regions is laryngeal cancer which presents a significant international health problem. This type of cancer has high rate of mortality because of the poor diagnosis in early stage of the disease. Despite favorable treatment in early-stage laryngeal cancers, survival rates for advanced-stage disease are less than 50%. Surgery and chemotherapy are two suitable treatment options that are used for laryngeal cancer. However, their combination is also is used. Recently the number of patients treated with radiotherapy and chemotherapy is increased (1). However, survival is decreased (2). Laryngeal cancer has been considered as a multifactorial disease associated with the interaction between environmental factors and genetic background (3). Environmental factors of laryngeal cancer are introduced as a lower consumption of vegetables and fruits, and higher consumption of milk, eggs, meat, tea, alcohol, and smoking (4). Recently, various studies have established the changes in molecular level which are associated with the development of laryngeal cancer. For example, several studies have investigated associations between CYP1A polymorphisms and laryngeal cancer risk (5). Alcohol consumption or smoking beside the uridine diphosphate glucuronosyl transferase enzyme (UGTs) rs4148323 act synergistically to increase the risk of laryngeal cancer (6). It has also reported the relationship between this type of cancer and nucleotide excision repair pathway genes such as ERCCs and XPA (7). The proteomics studies on laryngeal cancer show that the changed expression proteins regulate cellular proliferation, differentiation, and apoptosis that may directly related to the pathogenesis of cancer (8). Another one reported that some significantly changed expression proteins were the products of oncogenes and others were related to signal transduction and immune defense (9). Deeb A and colleagues showed that related DNA repair pathways are curtail in larynx cancer patients (10). For better understanding of molecular mechanisms of laryngeal cancer pathogenesis, protein-protein interaction (PPI) network analysis can provide an informative concept and detail schema (11-20). Therefore, we used a systems biology approach (based on the available proteomics literature data) as a rational strategy to reveal novel specific markers and probably therapeutic targets for laryngeal cancer. 

## Experimental

*Data collection*

In this study, the inclusion criteria were the studies on the human species using cell line and laryngeal squamous tissue samples involved in the comparison between the tumor and normal tissues. Exclusion criteria were the studies on non-human tissue and studies on samples of biological fluids, including plasma, serum, saliva, and urine. Studies only involved in comparison between the tumor tissue and tumor metastasis one. There was no limitation in methods in proteomic studies. We manually evaluated the publications in line with the above conditions; a total of 275 significantly changed expression proteinsextracted of which 136 proteins belong to up regulated protein group and 139 proteins were as down regulated proteins (See Tables 1 and 2).

**Table 1:** The list of up-regulated genes in tissue of human laryngeal cancer

**NO.**	**Gene name**	**NO.**	**Gene name**	**NO.**	**Gene name**	**NO.**	**Gene name**
1	ACAA1	35	EEF1D	69	HSPD1	103	PSMD2
2	ACTR2	36	EEF1G	70	IDH1	104	RAB2A
3	AKR1C2	37	EEF2	71	IMPDH2	105	RAP1B
4	ALB	38	EIF2S1	72	ISOC2	106	RPL14
5	ALDH3A1	39	EIF3F	73	KPNB1	107	RPL6
6	ANXA11	40	EIF3H	74	LAP3	108	RPS15A
7	ARHGAP1	41	EIF3I	75	LCP1	109	S100A16
8	ARHGDIA	42	EIF4A1	76	LDHB	110	S100A8
9	ARHGDIB	43	EIF5A	77	LGALS7	111	S100A9
10	ARL1	44	ENO1	78	LTA4H	112	SERPINB3
11	ARPC4	45	EPPK1	79	MAPRE1	113	SF3A3
12	ATIC	46	EPS8L1	80	METAP1	114	SFPQ
13	ATP6V1A	47	ERO1L	81	MPO	115	SND1
14	BLVRB	48	FABP5	82	MYL6	116	STAT1
15	C1QBP	49	FBP1	83	NAP1L1	117	TACSTD2
16	CA2	50	FLOT1	84	NCL	118	TAGLN2
17	CAND1	51	FN1	85	NDRG1	119	TALDO1
18	CAP1	52	FSCN1	86	NDUFA8	120	TAPBP
19	CAPN2	53	FTL	87	NP	121	TF
20	CAPNS1	54	FUS	88	PABPC1	122	TFRC
21	CCT6A	55	G3BP2	89	PDIA4	123	TKT
22	CCT7	56	G6PD	90	PDXK	124	TLN1
23	CDC37	57	GAPDH	91	PFN1	125	TPI1
24	CES1	58	GCN1L1	92	PGAM1	126	TPT1
25	CFL1	59	GFAP	93	PGK1	127	TRAP1
26	CLIC1	60	GNAI2	94	PGM1	128	TXNDC5
27	CMPK1	61	GSTP1	95	PLEC1	129	TYMP
28	COL12A1	62	HADHA	96	PLS3	130	USP14
29	CPSF6	63	HIST1H1B	97	PPA1	131	VASP
30	CTSB	64	HMGA1	98	PPP2R1A	132	VCL
31	CTSC	65	HNRNPA1	99	PRKRA	133	WARS
32	CYCS	66	HNRNPD	100	PRTN3	134	WDR1
33	DHX9	67	HNRPDL	101	PSMD11	135	XRCC5
34	ECH1	68	HSP90B1	102	PSMD13	136	YWHAZ

**Table 2:** The list of down-regulated genes in tissue of human laryngeal cancer

**NO.**	**Gene name**	**NO.**	**Gene name**	**NO.**	**Gene name**	**NO.**	**Gene name**	**NO.**	**Gene name**
1	A1BG	29	CORO1A	57	HIST1H1C	85	MYH11	113	RPS11
2	A2M	30	CORO1C	58	HNRNPL	86	MYH7	114	RPS15
3	ABHD14B	31	CRYAB	59	HP	87	MYL2	115	RPS9
4	ACADVL	32	CSTB	60	HSDL2	88	MYLPF	116	RRBP1
5	ACAT1	33	CTNND1	61	HSP90	89	NDRG2	117	SDHA
6	ACTG1	34	CYB5R3	62	HSPB1	90	NDUFA10	118	SERPINA1
7	AGR2	35	DCN	63	HSPG2	91	NDUFA12	119	SFN
8	AK3	36	DDOST	64	IARS2	92	NDUFS2	120	SLC4A1
9	ALDH2	37	DLD	65	IGHA1	93	OGDH	121	SOD1
10	ANXA2	38	DYNLL1	66	IGHG1	94	OGN	122	SOD3
11	APOA1	39	ECHS1	67	IGKC	95	ORM1	123	SP140
12	APOA2	40	EIF3A	68	IMMT	96	ORM2	124	SPTAN1
13	ASPN	41	EPHX1	69	ITIH2	97	PA2G4	125	SPTBN1
14	ATP5B	42	ERP29	70	JUP	98	PCYOX1	126	SSR4
15	ATP5D	43	EVPL	71	KRT19	99	PHB	127	TGFBI
16	ATP5F1	44	F13A1	72	LAMC1	100	PHB2	128	TMED10
17	ATP5O	45	FAU	73	LGALS3	101	PRDX3	129	TNNT3
18	BGN	46	FGB	74	LGALS3BP	102	PRELP	130	TPM1
19	C1QC	47	FGG	75	LMAN1	103	PSMB1	131	TRIM29
20	C3	48	FKBP4	76	LMAN2	104	PSME2	132	TROVE2
21	CALM1	49	GGT5	77	LMNA	105	PYCR1	133	U2AF1
22	CALML3	50	GLUD1	78	LMNB1	106	PYGB	134	UNC84B
23	CANX	51	GOT2	79	LRP1	107	RAN	135	UQCRB
24	CFH	52	GPD2	80	LTF	108	RPL10	136	UQCRC1
25	CFL1	53	GRP94	81	LUM	109	RPL19	137	UQCRC2
26	CKM	54	GSN	82	LYZ	110	RPL23A	138	VDAC1
27	CKMT1A	55	GSTP1	83	MARCKS	111	RPL9	139	VDAC2
28	COL15A1	56	H2AFY	84	MTPN	112	RPN1		

**Table 3:** Presentation of the hub proteins in the up-regulated and down-regulated protein–protein interaction networks of laryngeal cancer (top 20 in each PPI network). The hub nodes that play as bottleneck node are asterisked (for more details see Table 4 and discussion

	**ID**	**Degree**	**ID**	**Degree**	**ID**	**Degree**	**ID**	**Degree**
Up regulated	YWHAZ^*^	1634	CAND1*	827	PSMD2*	636	ALB*	524
	FN1*	1538	PABPC1	725	FUS*	631	NCL	508
	PPP2R1A*	1208	MAPRE1*	716	KPNB1*	618	STAT1*	503
	CDC37*	1158	HNRNPD*	703	DHX9	554	ACTR2*	492
	HNRNPA1*	1054	XRCC5*	661	EEF1G	538	CCT7	471
Down regulated	HSP90AA1*	2019	ACTG1*	681	RPL23A	449	LMNA*	407
	CALM3*	1276	P31947	569	CANX*	427	Q13813	390
	HSPB1*	1038	RPL9P9	484	P20618	424	PHB2	364
	RPL10*	992	RAN*	479	EIF3A	412	HNRNPL*	351
	DYNLL1*	792	RPS9	450	IGHG1*	411	U2AF1	348

**Table 4:** The list of top 20 up-regulated and down-regulated genes ranked based on BC from largest to smallest values

	**ID**	**BC**	**ID**	**BC**	**ID**	**BC**	**ID**	**BC**
Up regulated	PDXK	1.0	HNRNPA1	0.07400	FUS	0.04727	PSMD2	0.03599
	KHC	1.0	CDC37	0.06998	ENO1	0.04595	ALB	0.03178
	YWHAZ	0.13462	GNAI2	0.06835	HNRNPD	0.03861	HSPD1	0.03167
	FN1	0.13420	PPP2R1A	0.06310	ACTR2	0.03749	XRCC5	0.03007
	CAND1	0.07829	MAPRE1	0.04832	KPNB1	0.03667	STAT1	0.02821
Down regulated	HSP90AA1	0.20507	DYNLL1	0.06243	LGALS3	0.04051	APOA1	0.02707
	CALM3	0.13699	C3	0.06131	A2M	0.03737	IGHG1	0.02688
	HSPB1	0.07676	CANX	0.05931	RAN	0.03283	SOD1	0.02663
	ACTG1	0.07472	SFN	0.04720	FN1	0.03122	HNRNPL	0.02442
	RPL10	0.06626	LMNA	0.04078	PSMB1	0.02739	LGALS3BP	0.02210

**Table 5:** The modules of up regulated and down regulated PPI networks of human tissue of laryngeal cancer. The asterisked proteins are hub-bottleneck nodes

	**Category**	**MCODE score, nodes and edges**	**Seed**	**Hub**
Up regulated	Up-1	7.6, 65 and 358	NPM1	HNRNPD*, DHX9, FUS*, NCL and YWHAZ*
	Up-2	5.8, 65 and 320	HSPA9	KPNB1*, XRCC5* and CAND1*
	Up-3	4.0, 52 and 219	NS	PPP2R1A*
	Up-4	3.8, 49 and 115	----	HNRNPA1*
	Up-5	3.3, 13 and 44	----	ACTR2
Down regulated	Down-1	5.87, 65 and 219	UQCRC1	----
	Down-2	4.06 , 30 and 80	----	RPL9P9 ,DYNLL1*
	Down-3	4.0 , 15 and 43	----	----
	Down-4	4.0 , 10 and 30	----	CALM3*
	Down-5	4.0 , 18 and 80	----	ACTG1* , HSP90AA1*
	Down-6	3.25, 17 and 42	----	PHB2, U2AF1

**Table 6:** GO functional enrichment analysis of up- regulated and down-regulated PPI network modules. Top three terms of each module are tabulated

	**Category**	**Term**	**Description**
Up regulated	Up-1	GO:0006396	RNA processing
		GO:0000380	Alternative mRNA splicing
		GO:0071826	Ribonucleoprotein complex subunit organization
	Up-2	GO:0000082	G1/S transition of mitotic cell cycle
		GO:0042769	DNA damage response
		GO:1901992	Positive regulation of mitotic cell cycle phase transition
	Up-3	GO:0031398	Positive regulation of ubiquitination
		GO:0046364	Monosaccharide biosynthetic process
		GO:0006098	Pentose-phosphate shut
	Up-4	GO:0008380	RNA splicing
		GO:0022613	Ribonucleoprotein complex biogenesis
		GO:0031123	RNA 3 -end processing
Down regulated	Down-1	GO:0022904	Respiratory electron transport chain
		GO:0046034	ATP metabolic process
		GO:1902600	Hydrogen transmembrane transport
	Down-2	GO:1900739	Regulation of protein insertion into mitochondrial membrane involved in apoptotic signaling pathway
		GO:0031110	Regulation of microtubule (de) polymerization
		GO:0016259	Selenocystein metabolic process
	Down-3	GO:0010257	NADH dehydrogenase complex assembly
		GO:0006099	Tricarboxylic acid cycle

**Figure 1:**
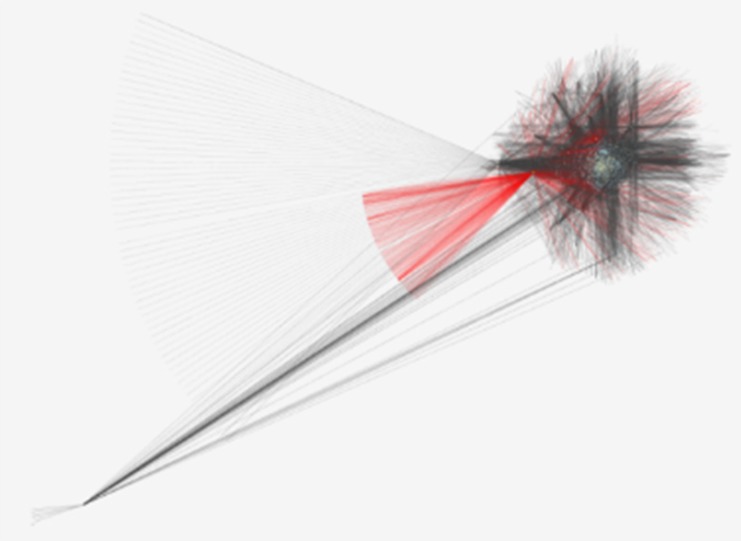
Protein-protein interaction network for up-regulated differentially expressed proteins in tissue of human laryngeal cancer include of 7312 nodes and 33757 edges

**Figure 2:**
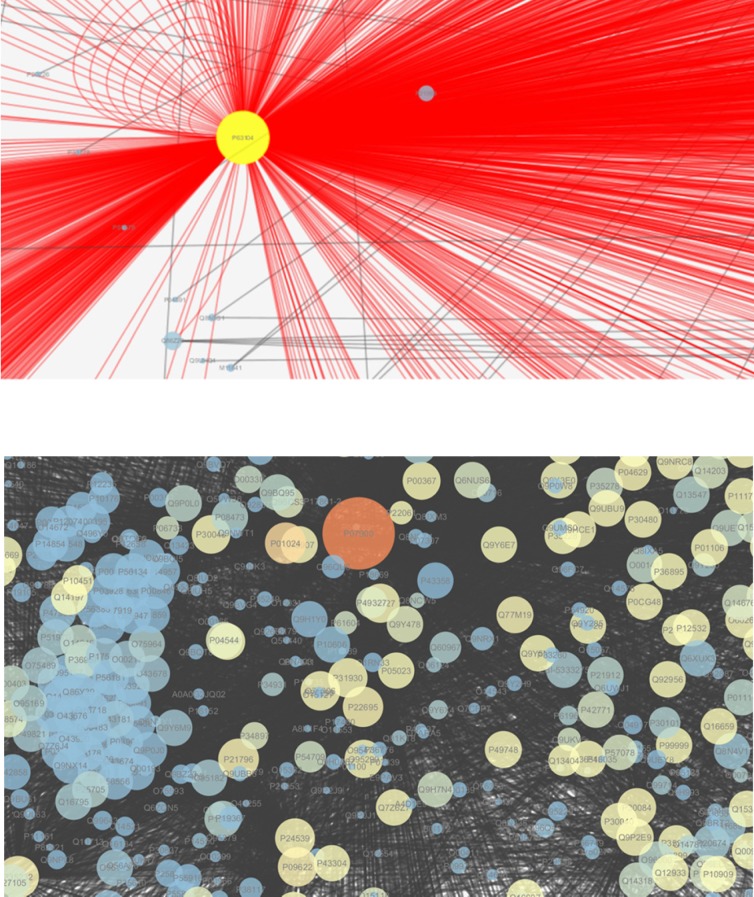
Up: Centrality analysis of protein-protein interaction network for down-regulated differentially expressed proteins in tissue of human laryngeal cancer consist of 6707 nodes and 27422 edges. Down: The dense and central part of upper network is shown in more details

**Figure 3:**
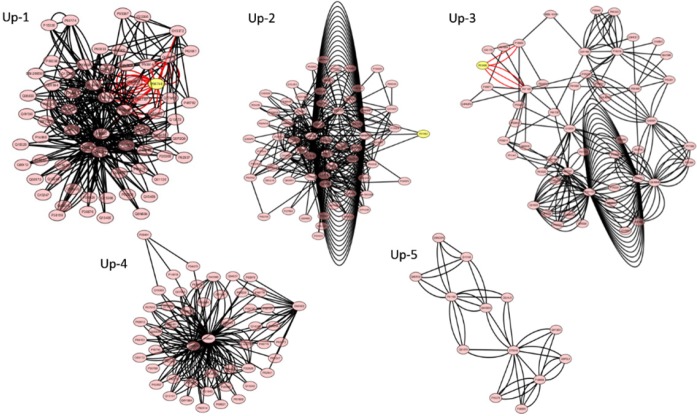
Modules of the protein-protein interaction network for up-regulated differentially expressed proteins (MCODE score > 3 and node > 6). The yellow cycles indicate seed proteins and the pink cycles reagent proteins in modules. There are no seed in Up-4 and Up-5 modules

**Figure 4:**
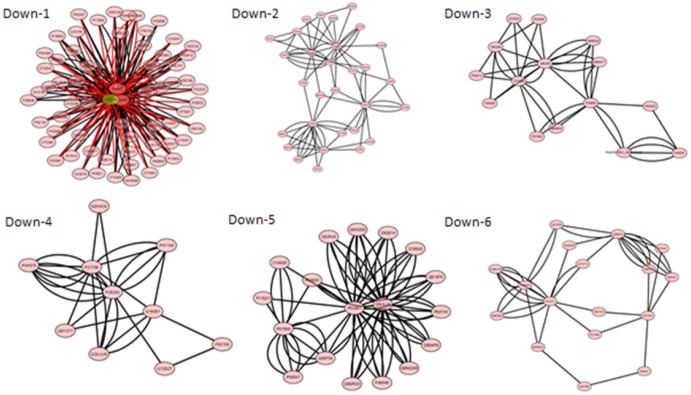
Modules of the protein-protein interaction network for down-regulated differentially expressed proteins (MCODE score > 3 and node > 6). The yellow cycles indicate seed proteins and the pink cycles reagent proteins in modules. Only Down -1 module has seed and the other ones have no seed

*PPI network analysis*

PPI network analyzed by Cytoscape Version 3.4 and Betweenness centrality (BC) and node degree the two major centrality parameters were analyzed by using a Cytoscape plug-in called ‘Network Analyzer’ (21). Degree indicates the number of connectivity belongs to a node and nodes having high degree were introduced as hub proteins. BC value the other centrality index reflects the shortest paths that pass through a node (22). 

*Screening of network modules and functional analysis*

The modules of the two constructed networks (including up and down regulated networks) were provided by MCODE analysis and parameters including Node Score Cutoff: 0.2, K-Core: 2, Degree Cutoff: 2 and, Max depth = 100 were used as the cut-off criteria for network module screening. MCODE score > 3 and node > 6 were considered for functional enrichment analysis of the modules. Kappa statistic ≥ 0.4 and Bonferroni step down method for probability value correction were used for annotation analysis of the selected modules.

## Results

After the submission of up-regulated and down-regulated proteins into Cytoscape, a total of 7312 and 6707 nodes related to the up-regulated and down-regulated proteins are included in the networks, respectively. In the final networks (Figures 1 and 2), the node›s degree was organized based on size; the nodes with high degree have bigger size and the blue to brown color represented low to high BC values for each node. \ The nodes with high degree were considered as key proteins. Then, the top 20 proteins with highest connectivity were identified as the hub proteins for each of the networks and similarly, the top 20 proteins based on betweenness centrality value were selected as bottleneck proteins (See Tables 3 and 4). 

*Module analysis*

A total of 11 modules including 5 up-regulated and 6 down-regulated network modules were obtained using default criteria. It was selected modules with MCODE score > 3 and node > 6. Five up-regulated modules (Up, 1-5) (Figure 3), and six down-regulated modules (Down, 1-6) (Figure 4) were selected for enrichment analysis.

There were some key proteins (hubs) in total of 5 up-regulated modules and 3 up-regulated network modules among them have 3 seed proteins (see Table 5). While, in down-regulated network modules, only Down-1 module has seed. The hubs in this network are distributed as tabulated data in Table 5.

*Functional enrichment analysis for modules*

Four up-regulated modules (Up, 1-4) and three down-regulated modules (Down, 1-3) were enriched based on functional annotation. The top three GO terms for each module are shown in Table 6.

## Discussion

Protein-protein interaction (PPI) network analysis has a significant growth in cancer studies to facilitate introducing early stage biomarkers (23). In our study, the laryngeal cancer related proteins were analyzed via PPI network construction, hub gene identification, module analysis, and functional enrichment analysis of most significant modules. These stages were carried out for up-regulated proteins and down-regulated ones in human laryngeal cancer tissue, separately. As it is shown in Tables 1 and 2, there are 275 changed expression proteins (including up and down regulated proteins) related to the human tissue of laryngeal cancer. Data management and analysis is a difficult process due to huge numbers of the collected proteins. Since PPI network analysis is a powerful method in categorization and ranking of the candidate and related proteins for a certain disease, here the up and down regulated networks are constructed separately (Figures 1 and 2). Topological analysis of the networks lead to rank of the nodes based on networks properties (18). By using two centrality indices including degree and betweenness, totally 80 nodes are selected among 275 initial proteins as important proteins (see Tables 3 and 4). However, the number of 80 nodes can not be considered as a suitable biomarker panel related to laryngeal cancer and more screening is required. The hub-bottleneck nodes for the up and down regulated networks are shown in Table 3. As it is shown in this Table there are 15 and 11 hub-bottlenecks for up and down regulated networks respectively. Module is a part of a network including closed related proteins havig specific biological function (20). Determined modules of network can provide informative perspective about different roles of the nodes (24). As it is shown in Figures 3 and 4 and Table 5 there are 5 and 6 modules for the up and down regulated networks respectively. Functional enrichment analysis for top score modules indicated that RNA processing and splicing, mitotic cell cycle regulation and sugar biosynthesis are affected by up-regulated modules while metabolic pathways and mitochondria are the main affected subjects by down regulated modules (see Table 6). The most significant pathways in four modules Up, 1-4 were RNA processing, G1/S transition mitotic cell cycle, protein ubiquitination and RNA splicing. It has been revealed overlapping between important pathways involved in the conversion of pre-mRNA to mature mRNA. In previous studies, it shows that polymorphisms of mRNA processing genes can be considered as risk factors for development of laryngeal cancer (25). The most significant pathways in down regulated modules (Down, 1-3) were respiratory electron transport chain, regulation of protein insertion in to mitochondrial membrane involved in apoptotic signaling pathway, and NADH dehydrogenase complex assembly. Proliferating cancer cells, such as laryngeal cancer, preferentially use anaerobic glycolysis rather than oxidative phosphorylation for energy production (26). In one system biology study, the glycolysis/gluconeogenesis pathway has been introduced as the most important pathway in laryngeal cancer (27). Then, the production of energy from mitochondrial respiratory may shift to glycolysis in laryngeal cancer. To prove this hypothesis and determine the energy supply sources of laryngeal cancer cells, more studies are needed. Regulation of protein insertion into mitochondrial membrane involved in apoptotic signaling pathway is the other important pathway in down regulated modules. One of the mechanisms impaired cancer cells is apoptosis. Apoptosis can be activated through several different signaling pathways, but a part of this mechanism is controlled in mitochondrial membrane through insertion apoptotic proteins (28). According to these results, in laryngeal cancer, apoptotic mechanism may disturb through the impairment of transporter proteins which transform apoptotic proteins into mitochondria. According the results of Table 5, the scattering of hubs in up-modules was more than down ones. Interestingly, the finding indicate that the seeds and hubs in up-modules have the similar functions with each other that are associated with regulation of cell cycle (29, 30). Among 26 hub-bottleneck nodes 12 proteins (8 up-regulated and 4 down-regulated proteins) are distributed in 8 modules (see Table 5). These proteins are tabulated in supplementary Table S1 and are ranked based on amounts of degree value. Here two suggestions are feasible: first investigation about expression changes of these 12 genes in the field and the second idea is selection of the top up and down regulated genes for more examinations. We choose cutoff 1200 for degree and therefore YWHAZ and PPP2R1A as the top two up-regulated genes and also HSP90AA1 and CALM3 as the top two down-regulated genes are introduced as human laryngeal cancer. YWHAZ gene with the highest degree and BC scores encodes 14-3-3 protein zeta/delta that has an essential role in tumor cell proliferation (31) through the regulation of multiple cellular processes, such as cell cycle control, anti-apoptosis, signal transduction, inflammation, and cell adhesion/motility (32). YWHAZ has been introduced as candidate proto-oncogene in head and neck squamous cell carcinoma whose reduced expression causes lower level of DNA synthesis rates (33). 14-3-3 proteins could be a key regulatory components in many processes that are crucial for development of cancers (34) such as laryngeal cancer (8). PPP2R1A gene encodes one subunit of protein phosphatase 2. This protein phosphatase is involved in control of cell growth and cell division processes. The role of this subunit in integrity of enzyme is highlighted. Therefore, it is expected that PPP2R1A plays a crucial regulatory role in cell proliferation in cancer cell line(35). HSP90AA1 and CALM3 were found as two top ranked genes in the down-regulated PPI network. These proteins belong to family of proteins which involved in the regulation of specific target proteins in cell cycle control and programmed cell death (36, 37). On the other hand, CALMs in addition to cell cycle, related to centrosome cycle and deregulation of this protein can be the origin of chromosomal instability in cancer (38). Interestingly, all determined possible biomarkers are related to the cell cycle process. 

## Conclusion

In this study, it has been represented a model of important proteins and pathways that provide a new level of information for laryngeal cancer that increases our knowledge about diagnostic and therapeutic aspects of this disease. Finally, a possible biomarker panel including YWHAZ and PPP2R1A as the two up-regulated genes and HSP90AA1 and CALM3 as the two down-regulated genes for human laryngeal cancer is introduced.
