# Supplementary material for: Introducing Potential Key Proteins and Pathways in Human Laryngeal Cancer: A System Biology Approach
Source: Iran J Pharm Res. 2018 Winter;17(1):415–25. (PMC5937111)
Supplement: Supplementary file 1 — Table S1 [file ijpr-17-415-s001.pdf]

Supplementary Materials for

Introducing Potential Key Proteins and Pathways in Human Laryngeal Cancer: A System Biology Approach

Hassan Peyvandi, Ali Asghar Peyvandi, Akram Safaei, Mona Zamanian Azodi and Mostafa Rezaei-Tavirani*

*To whom correspondence should be addressed. E-mail: tavirany@yahoo.com

Volume 17, Issue 1 (Winter 2018)

This PDF file includes:

Table S1

Table S1. The screened nodes based on degree, BC and module analysis. The nodes are ranked based on amounts of degree value.

	ID	Degree	BC	ID	Degree	BC
	YWHAZ	1634	0.13462	HNRNPD	703	0.03861
	PPP2R1A	1208	0.06310	XRCC5	661	0.03007
	HNRNPA1	1054	0.07400	FUS	631	0.04727
	CAND1	827	0.07829	KPNB1	618	0.03667
Down regulated	HSP90AA1	2019	0.20507	DYNLL1	792	0.06243
	CALM3	1276	0.13699	ACTG1	681	0.07472
